# Sequential maximum androgen blockade (MAB) in minimally symptomatic prostate cancer progressing after initial MAB: two case reports

**DOI:** 10.7497/j.issn.2095-3941.2014.04.007

**Published:** 2014-12

**Authors:** Mohan Hingorani, Sanjay Dixit, Fahim Bashir, Mohammad Butt, Simon Hawkyard, Richard Khafagy, Andrew Robertson

**Affiliations:** ^1^Department of Clinical Oncology, Castle Hill Hospital, Cottingham, HU16 5JQ, UK; ^2^Department of Urology, Scarborough General Hospital, Scarborough, YO12 6QL, UK

**Keywords:** Prostate cancer, castrate resistance, maximum androgen blockade (MAB)

## Abstract

The management of castrate-resistant prostate cancer progressing after maximum androgen blockade (MAB) has evolved in the last decade with the development of several novel therapeutic options. However, the initial therapeutic strategy in these patients usually involves withdrawal of anti-androgen that can be associated with biochemical response in approximately 20% of patients. Notably, we have observed evidence of sustained biochemical response in two patients following second- and third-line MAB using rechallenge schedule of previously administered anti-androgen after latent interval. The possibility of response following sequential MAB using the same anti-androgen agent has not yet been reported.

## Introduction

Androgen deprivation therapy using luteinizing hormone-releasing hormone (LHRH) analog or bilateral orchiectomy is the most common initial therapeutic strategy in patients with metastatic prostate cancer. The development of resistance to initial hormone manipulation is usually managed with maximum androgen blockade (MAB), which involves addition of an anti-androgen to suppress the androgen receptor (AR) signaling pathways. The management of prostate cancer progressing after initial (first-line) MAB often involves withdrawal of anti-androgen and consideration of palliative chemotherapy with docetaxel or other novel therapeutic drugs, such as abiraterone acetate, enzalutamide, and radium-223^1^.

Recently, increasing evidence suggests the use of second-line MAB with alternative anti-androgen therapy in prostate cancer progressing after initial MAB^2^^,^^3^. Anti-androgen withdrawal may also be associated with biochemical response in approximately 20% of patients. The optimum management of prostate cancer progressing after patients developed response to anti-androgen withdrawal remains poorly defined. We describe two patients who responded to bicalutamide withdrawal after first-line MAB and were rechallenged using subsequent lines of MAB with identical or alternative anti-androgen associated with sustained biochemical response. Based on our experience, the use of sequential MAB may be a valuable treatment approach for exploration before embarking onto more complex therapeutic strategies.

## Case reports

Patient demographics, disease characteristics, and outcomes after first and subsequent lines of therapies are summarized in **Table 1**. Both patients presented with advanced metastatic disease limited to bones and developed good response to initial hormone manipulation with LHRH analogue therapy or anti-androgen monotherapy (bicalutamide 150 mg OD) administered within licensed indications for management of advanced prostate cancer. Both patients received first-line MAB with addition of bicalutamide 50 mg OD after progression on initial LHRH analogue therapy. Subsequent management after progression on first-line MAB involved withdrawal of bicalutamide. Both patients had prolonged duration of response after first-line MAB ranging from 16 to 44 months and time to progression ranging from 13 to 46 months after withdrawal of initial anti-androgen therapy.

**Table 1:** Patient characteristics and sequential lines of therapy

Lines	Characteristics	
	Case 1	Case 2
Diagnosis	· 68 years	· 62 years
	· PSA =309 ng/mL	· PSA =1,300 ng/mL
	· Biopsy: Gleason 6 prostate AC	· Biopsy: Gleason 8 prostate AC
	· Multiple skeletal metastasis	· Multiple skeletal metastasis
	· 1^st^ line MAB: LHRH + bicalutamide 50 mg OD	· 1^st^ line: bicalutamide monotherapy 150 mg OD
	· PSA nadir =0.32 ng/mL	· PSA nadir =2.1 ng/mL
	· TTP =51 months	· TTP =18 months
First relapse	· PSA =19.3 ng/mL	· PSA =7 ng/mL
	· Bicalutamide withdrawal	· Bicalutamide stopped
	· PSA nadir = 0.32 ng/mL (at 6 months)	· patient commenced LHRH
	· TTP =46 months	· PSA nadir =1.3 ng/mL
		· TTP =12 months
Second relapse	· PSA =31.3 ng/mL	· PSA =14 ng/mL
	· 2^nd^ line MAB: LHRH + cyproterone 100 mg OD	· 1^st^ line MAB: LHRH + bicalutamide 150 mg OD
	· PSA nadir =7.5 ng/mL	· PSA nadir =1.3 ng/mL
	· TTP =30 months	· TTP =16 months
Third relapse	· PSA =37.1 ng/mL	· PSA =41 ng/mL
	· Cyproterone withdrawal	· bicalutamide withdrawal
	· PSA nadir of 19 ng/mL (at 4 months)	· PSA nadir =3.2 ng/mL (at 4 months)
	· TTP =15 months	· TTP =13 months
Fourth relapse	· PSA =48.6 ng/mL	· PSA =48.5 ng/mL
	· 3^rd^ line MAB: LHRH + bicaultamide 50mg OD	· 2^nd^ line MAB: LHRH + bicalutamide 50 mg OD
	· PSA nadir =33.1 ng/mL	· PSA nadir =20.1 ng/mL
	· TTP = not reached (>12 months)	· TTP =8 months
Fifth relapse	-	· PSA =55 ng/mL
		· Bicalutamide withdrawal
		· No response

PSA levels at the time of introduction of subsequent lines of MAB, ranged from 15 to 50 ng/mL with doubling time of more than 6 months. Both patients were minimally symptomatic without evidence of visceral metastasis. The first patient received second-line MAB using alternative anti-androgen in the form of cyproterone acetate, whereas the second patient was treated with second-line MAB using rechallenge schedule of bicalutamide 50 mg OD. The first patient also received third-line MAB with reintroduction of bicalutamide.

The use of subsequent lines of MAB was associated with >50% reduction in PSA levels and durable biochemical responses ranging from 8 to 30 months, but no objective radiographic responses were observed. The use of the above strategy delayed palliative chemotherapy for up to 21 months in one patient following development of castrate resistance after first-line MAB. The second patient still responds to third-line MAB at 91 months from development of castrate resistance after first-line MAB.

## Discussion

Anti-androgen withdrawal is a potential therapeutic strategy for patients with advanced prostate cancer progressing after initial MAB. Therapeutic responses have been documented following withdrawal of flutamide, bicalutamide, nilutamide, and estramustine. Such withdrawal underscores the presence of complex agonistic and antagonistic effects following interaction with intracellular ligand-regulated AR and modulation of downstream cellular transcriptional machinery. In a prospective study of 210 patients, anti-androgen withdrawal was associated with PSA response (>50% reduction in PSA level) in 21% of patients, in which 19% showed progression-free survival (PFS) of >12 months. However, no objective radiographic responses were observed. Patients with longer duration of initial therapy with MAB, with PSA levels of <10 ng/mL, and without radiographic evidence of metastasis were most likely associated with biochemical response following anti-androgen withdrawal^3^.

AR mutations and AR gene amplification are possible mechanisms for development of androgen resistance in MAB-exposed patients. Patients receiving MAB with flutamide showed increased incidence of mutation at codon 877 (threonine to alanine; T877A), and the presence of which has been associated with resistance to flutamide therapy related to loss of receptor inhibition and development of pro-stimulatory effects^4^. However, patients developing T877A mutations may still respond to exposure to alternative anti-androgen therapy with bicalutamide. Similarly, the use of bicalutamide has been associated with the development of mutation in codon 741 (tryptophan to cysteine; W741C); such mutation mediates resistance to bicalutamide, but W741C xenografts respond to flutamide, indicating that AR mutations may be drug-specific and non-cross resistant in nature^2^^,^^5^.

Previous studies have demonstrated therapeutic responses in patients with prostate cancer progressing after initial MAB following alternative anti-androgen exposure^6^^-^^8^. In a study of 232 patients with prostate cancer progressing after initial MAB, a change in the anti-androgen therapy (bicalutamide to flutamide or flutamide to bicalutamide) was associated with biochemical response in 62% of patients with improved survival^7^.

Both of our patients responded to reintroduction of same anti-androgen (bicalutamide) during subsequent MAB lines. On the one hand, the first patient was treated with alternative anti-androgen using cyproterone prior to rechallenge with bicalutamide at prolonged latency of 91 months between the two bicalutamide exposures. On the other hand, the second patient was retreated with bicalutamide at 13 months after initial exposure to the drug. These observations suggest the intriguing potential reversibility of AR mutations upon withdrawal of the specific anti-androgen drug, which may restore the response to the same agent when administered after latency. Whether intermittent anti-androgen therapy may be a more appropriate option for delaying the development of resistance in MAB patients also requires investigation.

**Figure 1:**
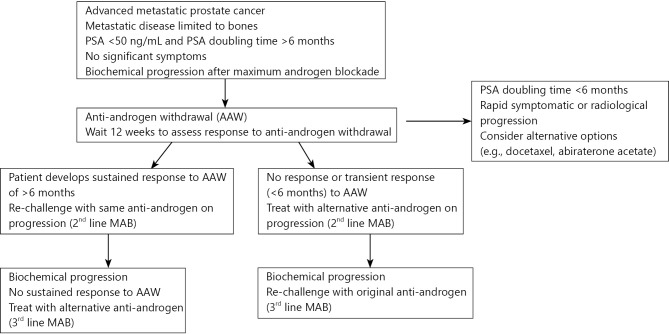
Probable hypothetical treatment algorithm for minimally symptomatic castrate-resistant cancer using sequential multiple lines of maximum androgen blockade.

The observations made in our patients support the possible use of subsequent MAB lines as a potential therapeutic strategy in patients developing response to first-line MAB, particularly in the context of anti-androgen withdrawal response. Although previous studies have demonstrated responses to alternative anti-androgen agents, our current observations provide the first evidence of the possibility of response following rechallenge with the same anti-androgen agent if prescribed after latency. Based on our experience, we have proposed a hypothetical treatment algorithm that may be used in patients with minimally symptomatic prostate cancer progressing after initial MAB depending on the anti-androgen withdrawal response (**Figure 1**). The proposed algorithm will require prospective validation before routine application in clinical practice.

In particular, the therapeutic strategy of sequential MAB elicits research attention for its cost effectiveness and excellent tolerance profile. This treatment may present a valuable alternative prior to the use of more complex therapeutic options, such as chemotherapy, abiraterone acetate, or enzalutamide, for the use of which may be sometimes limited because of inherent toxicity and economic constraints.
